# The 70- to 85-Year-Old Sweet Spot: Regression Associations Between Spiritual Experience and Successful Aging Outcomes

**DOI:** 10.1093/geroni/igaa057.1338

**Published:** 2020-12-16

**Authors:** Stephen Fogle

**Affiliations:** University of Nebraska Omaha, Omaha, Nebraska, United States

## Abstract

Utilizing the MIDUS III dataset, this study conducted linear regression analysis for associations between daily spiritual experience and Successful Aging (N=2605). Age was measured in three groups: young-old (55-69), old (70-85), and old-old (86-100). Successful Aging was operationalized as Self-Reported Physical Health, Self-Reported Memory, Depressed Affect, and Life Satisfaction. Daily spiritual experience was measured with the Fetzer Institute five-item composite scale (Cronbach = .891). Analysis for each Successful Aging outcome was controlled for daily spiritual experience, physical and social neighborhood environment, age group, gender identification, co-habitation, income, education, cultural identification and disability. Regression analysis was undertaken for daily spiritual experience on the same control variables. Results found higher frequency of daily spiritual experience was significantly associated (p = .000) with better self-reported memory (β= .146***) and higher life satisfaction (β= .191***). Further, regression analysis revealed the 70-85 age group was significantly associated (p = .000) with better self-reported physical health (β= .123***), lower depressed affect (β= -.144***), and higher life satisfaction (β= .291***). Finally, the 70-85 age group was a stronger predictor of daily spiritual experience (β= .221***) than all other control variables except female gender identification (β= .244***). This study contributes evidence of associations between daily spiritual experience and Successful Aging outcomes, particularly memory and life satisfaction. This study demonstrates the advantage of measuring separate old age categories to reflect heterogeneity of the life course. Finally, this study underscores, “Why Age Matters”, through new evidence linking the 70-85 year old age group with daily spiritual experience and Successful Aging.

